# Altered synthesis of genes associated with short-chain fatty acids in the gut of patients with atrial fibrillation

**DOI:** 10.1186/s12864-021-07944-0

**Published:** 2021-08-31

**Authors:** Jing Zhang, Kun Zuo, Chen Fang, Xiandong Yin, Xiaoqing Liu, Jiuchang Zhong, Kuibao Li, Jing Li, Li Xu, Xinchun Yang

**Affiliations:** Heart Center & Beijing Key Laboratory of Hypertension, Beijing Chaoyang Hospital, Capital Medical University, 8th Gongtinanlu Rd, Chaoyang District, 100020 Beijing, China

**Keywords:** Atrial Fibrillation, Short-chain fatty acids, Gut microbiota, Metagenomics

## Abstract

**Background:**

The gut microbiota provides health benefits in humans by producing short-chain fatty acids (SCFAs), whose deficiency causes multiple disorders and inflammatory diseases. However, gut bacteria producing SCFAs in patients with atrial fibrillation (AF), an arrhythmia with increasing prevalence, have not been reported. To investigate major gut microbial organisms related to SCFA synthesis, SCFAs-associated KEGG orthologues (KOs), enzymatic genes, and potential producers were examined according to metagenomic data-mining in a northern Chinese cohort comprising 50 non-AF control and 50 AF patients.

**Results:**

Compared with non-AF controls, individuals with AF had marked differences in microbial genes involved in SCFA-related synthesis, including 125 KOs and 5 SCFAs-related enzymatic genes. Furthermore, there were 10 species that harbored SCFA-synthesis related enzymatic genes, and were markedly decreased in the gut of AF patients. Notably, discriminative features about SCFA-synthesis related function, including 8 KOs (K01752, K01738, K00175, K03737, K01006, K01653, K01647 and K15023), 4 genes (*menI*, *tesB*, *yciA* and *CO dehydrogenase acetyl-CoA synthase complex*) and 2 species (*Coprococcus catus* and *Firmicutes bacterium CAG:103*), were selected as key factors based on LASSO analysis. Furthermore, PLS-SEM analysis showed that 72.8 and 91.14 % of the overall effects on gut microbiota diversity and key species on AF, respectively, were mediated by the key KOs. Meanwhile, 46.31 % of the total effects of SCFA-synthesis related function on left atrial enlargement was mediated by hsCRP. Upon incorporation of clinical properties in AF, the KO score was still significantly associated with AF incidence (OR = 0.004, P = 0.001).

**Conclusions:**

The current study revealed that dysbiotic gut microbiota in AF is coupled with disrupted SCFA-synthesis related genes, characterized by decreased abundances of KEGG orthologues, synthesis enzymatic genes and harboring species.

**Supplementary Information:**

The online version contains supplementary material available at 10.1186/s12864-021-07944-0.

## Background

Atrial fibrillation (AF), a major arrhythmia, is associated with high morbidity and mortality. Over the past several years, significant advances in AF therapy have been made, but there is certainly plenty of room for improvement in disease management, e.g., to alleviate the by-effects of anti-arrhythmia drugs and recurrence post-catheter ablation [1]. Therefore, a deep understanding of the potential basis of AF development and progression is urgently required.

Communications between the gut microbiota and the host organism play pivotal roles in diverse diseases. We have previously descripted the profile of dysregulated gut microbiota in AF [2]. Dysbiotic gut microbiota already occurs in the early stage of AF, likely constituting an early disease regulator and potential factor to delay AF progression [3, 4]. Meanwhile, the altered gut microbiota profile might have a clinical value in predicting AF recurrence after catheter ablation [5]. Although the disordered gut microbiota in AF has been characterized, the underlying mechanisms remain elusive.

Among the numerous metabolites produced by gut microbes, short-chain fatty acids (SCFAs) have been reported as key bacterial metabolites with critical roles in regulating inflammation and immune homeostasis. SCFAs, including acetic, propionic and butyric acids, are biosynthesized by microorganisms through fermentation of dietary fibers; they are directly involved in G-coupled-receptor activation and histone deacetylase inhibition, and represent energy substrates, thus affecting multiple physiological events and likely contributing to human health [6]. A large body of evidence has demonstrated that deficiency in SCFAs leads to diseases such as hypertension and related cardiovascular disease, type 2 diabetes mellitus and obesity [7–9]. However, relevant features of gut-derived SCFA production in individuals with AF remain undefined, as well as whether SCFAs constitute a pathological link between altered gut microbiota and AF.

Therefore, the current study carried out mining of metagenomic sequencing data to evaluate the profile of bacterial genes related to SCFA-biosynthesis enzymes, examine the bacterial functions of SCFA-related synthesis, and determine gut species harboring these enzymatic genes and functions in individuals with AF. Moreover, the interaction between corresponding bacteria, enzymatic genes, and microbial metabolic pathways critical to SCFA-related synthesis might provide new mechanistic insights for gut microbiota dysbiosis and concomitant SCFA-related disorders in the gut of individuals with AF.

## Results

### Functions related to SCFA-synthesis in the gut of AF cases

First, we reviewed the functions related to SCFA-synthesis in the gut microbiota of individuals with AF based on Kyoto Encyclopedia of Genes and Genomes (KEGG) orthologues. The microbial transformation of dietary fibers in the intestine helps synthesize three main SCFAs such as acetic, propionic and butyric acids. Acetic acid production uses pyruvate via acetyl-CoA. Butyric acid is biosynthesized from two acetyl-CoA molecules that yield acetoacetyl-CoA, which is subsequently transformed into butyryl-CoA. Propionate can be formed from lactate by reduction [6] and produced from succinate [10]. In addition, isobutyric acid, valeric acid, isovaleric acid, isocaproate, and 2-methylbutyrate were SCFAs presented with branched-chain conformation, which were classified as branched-chain fatty acids (BCFAs) [11].

**Fig. 1:**
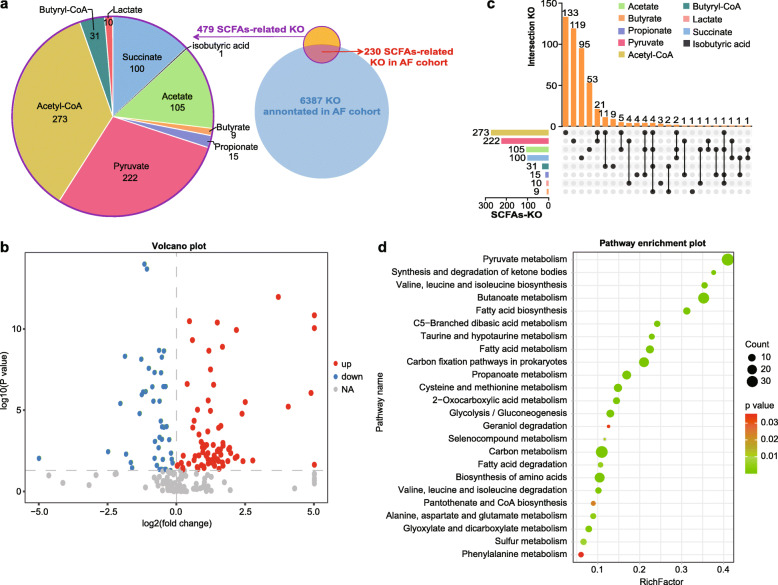
SCFA-related KOs in the gut of AF patients. **(a)** Venn diagram showing a total of 479 KEGG orthologues (KOs) that were involved in seven short-chain fatty acids (SCFAs)-related compounds (left panel), while 230 KOs overlapped among the 6387 annotated KOs in the current cohort (right panel). **(b)** Volcano plot displaying 125 different KOs between atrial fibrillation (AF) patients and non-AF controls (red and blue denote significant enrichment and decrease in AF, respectively; grey indicates a non-significant difference between the two groups). **(c)** Distributions of KOs in various SCFAs-related compounds. The orange bar chart above represents the numbers of KOs contained in various compounds. The bar chart at the bottom left represents the numbers of KOs included in various compounds (a different color was used for each compound). The dotted line at the bottom right shows the intersected KOs contained in each compound. Wilcoxon rank-sum test q < 0.05 indicated statistical significance (Benjamini-Hochberg correction was carried out to determine q values). **(d)** Bubble chart of 24 KEGG pathways related to the 125 differential KOs

According to the retrieved results from the acetate (C00033), butyrate (C00246), propionate (C00163), pyruvate (C00022), acetyl-CoA (C00024), butyryl-CoA (C00136), lactate (C00186), succinate (C00042), isobutyric acid (C02632), valeric acid (C00803), isovaleric acid (C08262), isocaproate (C21399), and 2-methylbutyrate (C18319), 479 KOs participating in the bioprocess of SCFAs were categorized as SCFAs-related KOs (Supplementary Table S1). Among the total of 6387 KOs annotated in the current cohort, 230 were mapped into SCFA-related KOs assoiated with AF (Fig. 1a).

Next, we compared the abundance levels of 230 SCFA-related KOs across non-AF controls and AF patients to identify the enrichment of deficient ones in AF. Overall, 125 SCFA-related KOs showed marked differences between non-AF controls and AF patients (*P* < 0.05 in Wilcoxon rank sum test with Benjamini-Hochberg correction, Fig. S1). While 81 KOs were enriched in AF, 44 were deficient (Fig. 1b). The distribution of the 125 KOs for SCFAs-related compounds is shown in Fig. 1c. Specifically, the various KOs were mapped into KEGG pathways such as pyruvate metabolism, fatty acid biosynthesis, butanoate metabolism and propanoate metabolism (Fig. 1d).

### Enzymatic genes related to SCFAs-synthesis in gut of AF patients

**Fig. 2:**
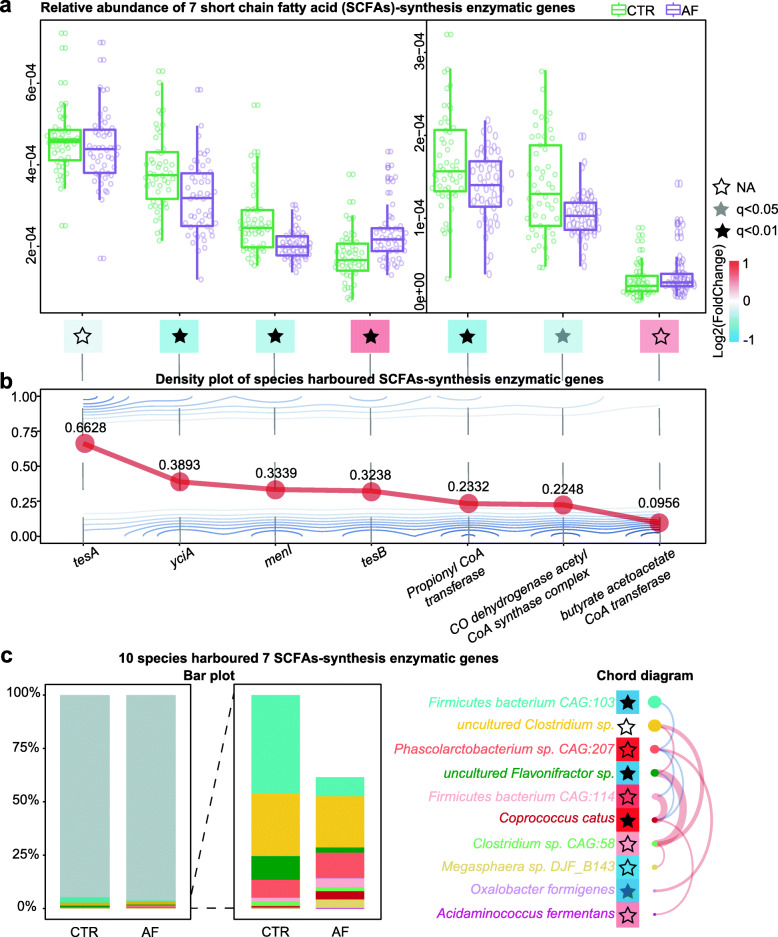
SCFA-related synthetic enzyme genes and harboring species in the gut of AF patients. **(a)** Box plots showing the relative abundance levels of seven short-chain fatty acids (SCFAs)-related synthetic enzyme genes. Boxes reflect interquartile ranges; lines and circles depict medians and outliers, respectively. The color of the block under each box denotes enrichment assessed by Log2 (Fold Change), with red and blue indicating enrichment in the atrial fibrillation (AF) cases and non-AF controls (CTR), respectively. The color of the star inside the block indicates the q value (q < 0.01, dark gray asterisk; q < 0.05, light gray asterisk; q ≥ 0.05, transparent asterisk; N.S., not significant;). **(b)** Density plot showing the relative number of species that harbored SCFA-related enzyme genes. **(c)** Stack bar plot depicting the relative abundance levels of the 10 species that harbored synthesis enzyme genes for SCFAs-related compounds; the color of the bar reflects a given species. The right panel is a chord diagram showing the links among the 10 species with spearman correlation |r|>0.15, where red and blue lines denoting positive and negative correlations, respectively. The blocks and stars have the same descriptions as in panel (a)

Then, specific bacterial genes coding for enzymes involved in SCFA-related synthesis were investigated. Previous studies have indicated that bacterial genes such as *yciA*, *tesA*, *tesB*, and *menI*, as well as *propionyl CoA transferase*, *CO dehydrogenase acetyl-CoA synthase complex*, and *butyrate acetoacetate CoA transferase* can contribute to SCFA-related formation [12, 13]. The majority of microbial genes for SCFA-related synthesis enzymes were starkly decreased in the intestine of individuals with AF (q > 0.05, Log 2 [Fold Change] = -0.0653 for *tesA*; q = 9.96E-05, Log 2 [Fold Change] = -0.3025 for *yciA*; q = 1.33E-03, Log 2 [Fold Change] = -0.2359 for *menI*; q = 6.37E-03, Log 2 [Fold Change] = -0.3311 for *propionyl CoA transferase*; q = 1.90E-02, Log 2 [Fold Change] = -0.1618 for *CO dehydrogenase acetyl-CoA synthase complex*; Fig. 2a). In addition, *tesB* (q = 1.18E-04, Log 2 [Fold Change] = 0.3819) and *butyrate acetoacetate CoA transferase* (q > 0.05, Log 2 [Fold Change] = 0.2828) levels were elevated in AF, although with moderate differences and low abundance levels (Fig. 2a).

Furthermore, 11 enzymatic genes associated with BCFAs were investigated based on the pathway retrieved from KEGG database (Fig. S2a), with 5 genes significant decreased in the gut of AF individuals (q = 5.38E-08, Log 2 [Fold Change] = -0.4844 for *3-methyl-2-oxobutanoate dehydrogenase*; q = 0.0002, Log 2 [Fold Change] = -0.2364 for *acetolactate synthase*; q = 0.0498, Log 2 [Fold Change] = -0.0940 for *2-oxoisocaproate dehydrogenase*; q = 0.0230, Log 2 [Fold Change] = -0.1333 for *3-isopropylmalate dehydratase*; q = 1.05E-05, Log 2 [Fold Change] = -0.3674 for *dihydrolipoyl dehydrogenase*).

### Gut species harboring enzyme genes related to SCFAs-synthesis in AF

Intestinal bacterial organisms harboring enzyme genes related to SCFAs-synthesis in AF cases were determined. The enzyme genes for the SCFAs-related compounds were aligned to the integrated nr database for evaluating taxonomic allocation. In this study, 596 species harbored at least 1 SCFA-synthesis related enzymatic gene. Precisely, 395 species harbored *tesA*, 232 harbored *yciA*, 199 harbored *menI*, 193 harbored *tesB*, 139 harbored *propionyl CoA transferase*, 134 harbored *CO dehydrogenase acetyl CoA synthase complex* and 57 harbored *butyrate acetoacetate CoA transferase* (Fig. 2b).

Importantly, the abundance levels of 10 species, including *Firmicutes bacterium CAG:103, uncultured Clostridium sp., Phascolarctobacterium sp. CAG:207, uncultured Flavonifractor sp., Firmicutes bacterium CAG:114, Coprococcus catus, Clostridium sp. CAG:58, Megasphaera sp. DJF_B143, Oxalobacter formigenes* and *Acidaminococcus fermentans*, which harbored synthesis genes for SCFAs-related compounds were markedly decreased in the gut of individuals with AF (Fig. 2c**)**. For instance, *Firmicutes bacterium CAG:103* and *uncultured Flavonifractor sp.* were remarkably decreased in AF patients. Also, *uncultured Flavonifractor sp.* was positively correlated with *uncultured Clostridium sp.* and *Clostridium sp. CAG:58*, and negatively associated with *Coprococcus catus* (Table S2). The complex links among these species harboring SCFAs-related synthesis enzyme genes indicated the disordered profile of SCFA-related producers in AF patients. Meanwhile, 11 species harboring 10 BCFAs-encoding genes were decreased in gut of AF patients, and half of them were overlapped with SCFAs-related species, such as *uncultured Clostridium sp.*, *Coprococcus catus*, *Oxalobacter formigenes*, *Firmicutes bacterium CAG:103*, and *Firmicutes bacterium CAG:114* (Fig. S2b, c).

### Key gut SCFA-synthesis related factors selected by LASSO analysis

**Fig. 3:**
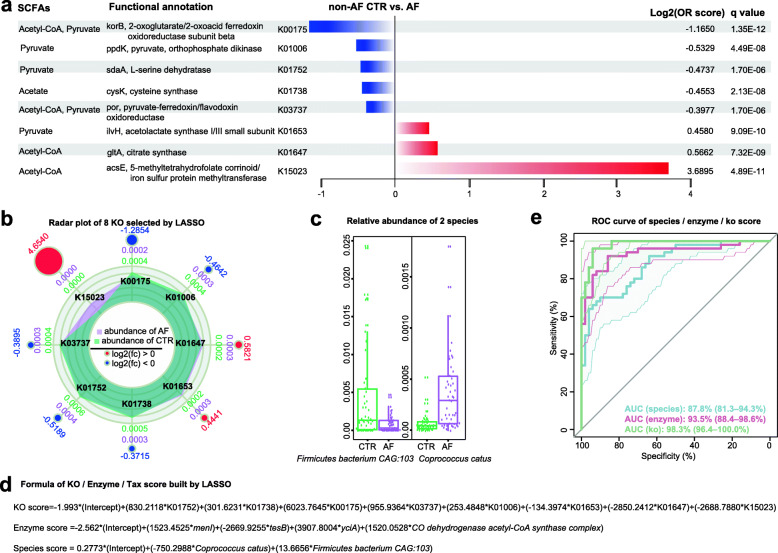
Key gut SCFA-related synthesis factors selected by LASSO analysis. **(a)** Relative abundance levels of the 8 KEGG orthologues (KOs) selected by the least absolute shrinkage and selection operator (LASSO) analysis from different KOs between non-AF controls and AF patients. Log 2 (Odds ratio [OR] score) < 0 (decreased in AF, blue); Log 2 (OR score) > 0 (enriched in non-AF controls, red). **(b)** Radar plots of the 8 KOs selected by LASSO analysis. Green and purple denote the relative abundance levels of atrial fibrillation (AF) and non-AF controls (CTR), respectively. The outer circle reflects the fold change, with red and blue indicating enrichment in AF and control patients, respectively. **(c)** Box plot of 2 species selected by LASSO analysis. **(d)** Formulae of KO, enzyme and species scores obtained by LASSO analysis. **(e)** Receiver operating curves for KO, enzyme and species scores in AF

Next, we aimed to establish a model that reflects the SCFA-synthesis related function in individuals. Firstly, we selected key factors from different SCFA-related KOs, enzyme genes, and related species in non-AF controls and AF patients by LASSO analysis. We found that 8 KOs (Fig. 3a, b), 4 genes and 2 species (Fig. 3c) among the candidate variables remained statistically significant, with nonzero coefficients (Fig. S3).

To assess the differential enrichment of specific KOs in AF patients versus non-AF control cases, Log 2 (odds ratios [ORs]) were determined. An ortholog or module with Log 2 (OR) > 0 was considered to be enriched in AF, while that with Log 2 (OR) < 0 was categorized as CTR enriched. Most of the key SCFAs-related KOs were decreased in AF, including K00175, mapped into the reaction of R01196 (2 Reduced ferredoxin + Acetyl-CoA + CO2 + 2 H^+^ <=> 2 Oxidized ferredoxin + Pyruvate + CoA). Moreover, a few KOs were relatively enriched in AF, including K15023 (mapped into the reaction of R10243 [Tetrahydrofolate + Acetyl-CoA <=> 5-Methyltetrahydrofolate + CoA + CO]), K01647 (mapped into the reaction of R00351 [Citrate + CoA <=> Acetyl-CoA + H2O + Oxaloacetate]) and K01653 (mapped into the reaction of R00226 [S-2-Acetolactate + CO2 <=> 2 Pyruvate]), with both acetyl-CoA and pyruvate being precursors of SCFAs (Fig. 3a, b). Therefore, we speculate that deficiency in key enzymes, such as *yciA*, *tesB* and *menI*, which catalyze the process from SCFA precursors to SCFAs, caused excessive precursor accumulation.

Then, KO, enzyme and species scores were defined based on linear combinations of selected factors and respective coefficients. The models were constructed as follows: KO score = -1.993 * (Intercept) + (830.2118 * K01752) + (301.6231 * K01738) + (6023.7645 * K00175) + (955.9364 * K03737) + (253.4848 * K01006) + (-134.3974 * K01653) + (-2850.2412 * K01647) + (-2688.7880 * K15023); Enzyme score = -2.562 * (Intercept) + (1523.4525 * *menI*) + (-2669.9255 * *tesB*) + (3907.8004 * *yciA*) + (1520.0528 * *CO dehydrogenase acetyl-CoA synthase complex*); Species score = 0.2773 * (Intercept) + (-750.2988 * *Coprococcus catus*) + (13.6656 * *Firmicutes bacterium CAG:103*) (Fig. 3d).

To evaluate the specific value of the SCFA-synthesis related function model, the area under the ROC curve (AUC) was assessed and compared with those obtained for these scores. Notably, AUCs for the KO (AUC = 0.983, 95 %CI: 0.964-1, *p* = 8.73E-17), enzyme (AUC = 0.935, 95 %CI: 0.884–0.986, *p* = 6.70E-14) and species (AUC = 0.878, 95 %CI: 0.813–0.943, *p* = 7.29E-11) score models were relatively high (Fig. 3e).

### Interactions among gut microbial organisms, SCFA-synthesis related enzyme genes, bacterial functions, and potential producers of SCFAs in AF

**Fig. 4:**
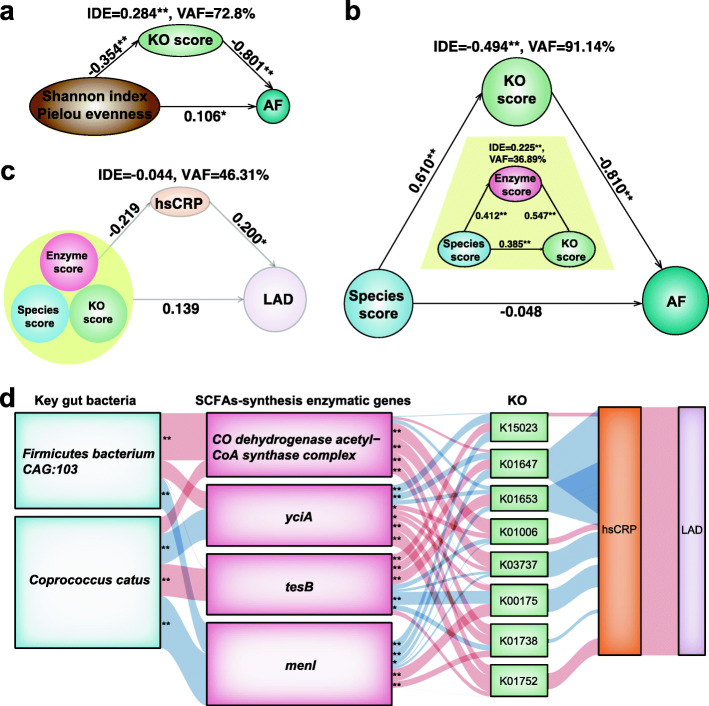
Interactions among gut microbes, SCFA-related synthetic enzyme genes, bacterial functions, and putative producers of SCFAs in AF. **(a-c)** The partial least squares structural equation modeling (PLS-SEM) models showing the mediation effect of short-chain fatty acids (SCFAs)-related synthetic function in the total effect of gut microbiota diversity (**a**) and key bacteria (**b**) in atrial fibrillation (AF), and the mediation effect of potential systematic inflammation reflected by high-sensitive c reactive protein (hsCRP) in the total effect of SCFA-related synthetic function in left atrial enlargement in AF (**c**). Path coefficients are indicated; indirect effect and variance accounting for the score are found below a given mediator (*P < 0.05; **P < 0.01). **(d)** Interrelationship of the SCFA-related synthesis potential of different bacteria. The correlation network obtained by Spearman correlation analysis depicts key intestinal bacteria with significant associations with indicated SCFA-related synthetic enzyme genes, linked to respective KEGG orthologues (KOs). Red and blue indicate positive and negative correlations, respectively; line thickness reflects correlation strength. FDR corrected *p* < 0.05 (*) or 0.01 (**) was considered statistically significant

Considering the correlation between gut microbiota dysbiosis and inflammation, both of which are involved in AF, we formulated the hypothesis that dysbiotic gut microbiota in AF development occurs via SCFA-related deficiency derived systemic inflammation. Therefore, we applied partial least squares structural equation modeling (PLS-SEM) to assess potential mediating effects (indirect effects, IDEs) of SCFAs during gut microbiota shift in individuals with AF. Firstly, we determined the variance accounted for (VAF) score, ratio of indirect-to-total effect determining the proportion of the variance due to mediation. The VAF for disrupted SCFA-synthesis related function reflected by the KO score was 72.8 % (elevated gut microbiota diversity, Fig. 4a) and 91.14 % (species score, Fig. 4b) while that of the enzyme score was 36.89 % (Fig. 4b). Notably, the VAF for high-sensitive c reactive protein (hsCRP), a marker commonly associated with systematic inflammation, [14] during the process of left atrial diameter enlargement mediated by disordered SCFA-synthesis related function was 46.31 % (Fig. 4c). Moreover, the influence of AF comorbidities on the hsCRP level was assessed, and the results showed that hsCRP level is not significantly different in AF patients with or without the comorbidities of hypertension (p = 0.0991) or diabetes millitus (p = 0.1606). Thus, the PLS-SEM results suggested that the involvement of gut microbiota dysbiosis in AF was partially influenced by the altered SCFA-related synthesis, while SCFA-related deficiency might contribute to atrial remodeling through inflammation.

For evaluating the SCFA-related synthesis potential of bacteria in AF cases, correlation analyses among these metrics were carried out. The results revealed that the key SCFA-related producers *Firmicutes bacterium CAG:103* and *Coprococcus catus* were significantly associated with *CO dehydrogenase acetyl-CoA synthase complex*, *yciA*, *tesB, menI*, and 8 SCFA-related KOs, and further correlated with hsCRP and left atrial diameter (Fig. 4d).

In addition, to evaluate whether the SCFA-related synthesis function represented by the KO score is a conventional risk factor [15] compared with clinical characteristics, univariable and multivariable logistic regression analyses were carried out, assessing ORs and corresponding 95 %CIs for factors associated with AF. We found that age, body mass index and KO score (OR = 0.012, 95 % CI: 0.002–0.086; *P* = 1.1E-05) were significantly associated with AF. Multivariate-adjusted logistic regression was performed taking into account the abovementioned parameters. Upon incorporation of the clinical features of AF cases, the KO score was still significantly associated with AF incidence (OR = 0.004, 95 %CI: 1.54E-04–0.097, *P* = 0.001). Thus, the KO score independently predicted AF occurrence (Table S3).

## Discussion

Although the gut microbiota-derived metabolites SCFAs contribute to the alleviation of various inflammation-mediated ailments, such as diabetes mellitus [7], multiple sclerosis [16], gut homeostasis [17], graft-versus-host disease [18], and cardiovascular disease [19], studies depicting the role of the gut microbiota in SCFA synthesis in AF are scarce. AF, the commonest cardiac arrhythmia with an increasing prevalence worldwide, is frequently associated with enhanced inflammatory response [20]. Our previous studies have characterized the dysbiotic gut microbiota profiles of AF patients with different phenotypes. In the present study, we assessed the profiles of SCFA-related synthesis function and potential bacteria producing SCFAs in AF patients on the basis of metagenomic data. In addition, further description of the links among SCFA-related synthesis function and AF-correlated indicators, including left atrial diameter and hsCRP, was provided.

Mounting evidence indicates that the complex relationships among prebiotic fibers, gut bacteria and SCFAs constitute a key modulator of inflammatory disorders. The harmful impact of low-fiber Westernized diets might be responsible for hypertension, via insufficient SCFA production and GPR43/109A signaling. The cardioprotective effects of SCFAs are modulated by the cognate SCFAs receptors GPR43/GPR109A, as well as DNA methylation-modulated T regulatory cell amounts [8]. Another study also reported that the beneficial anti-inflammatory properties limiting cardiovascular disease progression via propionate effects are mainly dependent on regulatory T cells and T helper cell homeostasis, thereby reducing cardiac hypertrophy and fibrosis, susceptibility to cardiac arrhythmias, and atherosclerotic lesion burden [19]. Propionic acid could shape multiple sclerosis disease course by an immunomodulatory mechanism involving stimulation of Treg cells, and exert direct neurodegenerative effects, linking the gut metabolome to the brain-immune axis [16]. Bacteriotherapy by the replenishment of youthful gut microbiome containing higher SCFA levels and related SCFA-producing strains could reverse poor stroke recovery in aged mice [21]. These reports suggested that maintaining a healthy, SCFA-synthesizing microbiome is critical to health, indicating that the associations of gut bacteria with SCFA synthesis pathways in AF should be further investigated.

Meanwhile, there is plausible evidence linking inflammation to the initiation and perpetuation of AF [22]. The current study discussed preliminarily about the underlying mediation effect of gut microbiota-based SCFAs-related function on AF as well as the potential influence on left atrial enlargement, and the results from PLS-SEM reflected the potential correlation of hsCRP, a inflammatory marker. We speculated the proposed mechanisms linking SCFA-deficiency related-inflammation and AF might include the inhibitory effects of NACHT, LRR and PYD domain containing protein 3 (NLRP3) inflammasome. The pathophysiological function of NLRP3-inflammasome signaling in cardiomyocytes (CMs) with a mechanistic link to AF pathogenesis has been well established. NLRP3-inflammasome activity is elevated in atrial CMs in individuals with paroxysmal and long-standing or persistent AF, and CM-specific knock-in (KI) mice producing constitutively activated NLRP3 exhibit ectopic activity, altered sarcoplasmic-reticulum Ca^2+^ release, shortened atrial effective refractory period, and atrial hypertrophy [23]. Previous evidence shows that acetic acid inhibits inflammasome activation through the Gq/11 subunit of G-protein coupled-receptor-43 (GPR43) in a Ca^2+^-dependent manner by subsequent phospholipase C-inositol triphosphate signaling, further activating soluble adenylyl cyclase, promoting NLRP3 inflammasome ubiquitination by protein kinase A, and finally inducing NLRP3 degradation via autophagic pathways [24].

Recently, synthetic biologists are working at the level of engineering gut bacteria to deliver therapeutic payloads [25]. For example, an engineered strain of *Escherichia coli Nissle 1917* with insertion of the genes encoding phenylalanine ammonia lyase and L-amino acid deaminase into the genome, could allow for bacterial consumption of phenylalanine within the gastrointestinal tract for the treatment of phenylketonuria [26]. An engineered strain *Escherichia coli BL21* equipped with high β-galactosidase activity could play a role in lactose intolerance alleviation [27]. Furthermore, transplantation of defined microbial communities with genetically human commensals with engineered microbial *cutC* gene (an enzymatic source of choline-to-trimethylamine transformation) into germ-free mice is sufficient to transmit trimethylamine-N-oxide production, heighten cerebral infarct size, and lead to functional impairment [28]. And for that, transplantation of engineered bacteria equipped with upregulated-SCFA synthetic function, such as some decreased SCFA-synthesis enzymatic genes in AF as described in the current study, might contribute to SCFA production, and play a beneficial regulatory role in disease progression. Moreover, the beneficial effect that modulating microorganisms supplementing dietary fiber to replenish disease-decreased SCFAs has been revealed in some disease such as stroke [21], multiple sclerosis [16], and hypertensive cardiovascular disease [19]. These extensive findings will pave the way to translate gut microbiota use for clinical intervention, and more studies are imperative to evaluate its clinical value in the context of AF.

Yet, the present study had some limitations. Targeted metabolomic analysis of SCFAs was not carried out because specimen collection was complex. Therefore, actual abundance levels of SCFAs in AF patients could not be obtained, and we were unable to validate the interactions among KO, enzymatic genes, and harbored bacterial strains. The determination of SCFAs level in AF populations as well as mice colonized with SCFA-targeted engineering bacteria might have value, which will be explored in our future work. Notably, the substances of SCFAs are not distributed homogeneously in the contents of large intestine, as well as in circulation at portal, hepatic or peripheral venous blood [29]. So the level of SCFAs varies greatly depending on where the sample is taken, which increasing the heterogeneity of measurement. Therefore, the genes coding for SCFAs based on the metagenomic data exhibited higher temporal stability.

## Conclusions

This study revealed the profiles of genes associated with SCFA-synthesis in the intestine of individuals with AF on the basis of data-mining of taxonomic makeup and bacterial functions from the metagenome. Dysbiotic gut microbiota detected in the AF state was coupled with disordered SCFA-synthesis related function, characterized by decreased abundance levels of SCFA-related KOs, synthesis enzyme genes and harboring species.

## Methods

### Study cohort

Metagenomic sequencing data of 50 nonvalvular AF patients and 50 control individuals from northern China were analyzed from a previous trial by our team [2], where the sample size has been evaluated as sufficient according to the analysis of rarefaction curve. Exclusion criteria included previous cardiac dysfunction; coronary artery disease; comorbidities, including autoimmune ailments, and liver or kidney dysfunction; cancer; use of antibiotics or probiotics less than one month pre-enrolment. AF was diagnosed by electrocardiography. The baseline characteristics of the 100 individuals were shown as supplementary Table S4. The study had approval from the ethics committees of Beijing Chaoyang Hospital and Kailuan General Hospital. Signed informed consent was provided by each participant.

### Analyses of SCFA-synthesis related KOs, enzyme genes and harboring species based on metagenomic data

Freshly collected fecal specimens underwent bacterial DNA extraction with a TIANamp Stool DNA Kit (DP328, TIANGEN Biotech, China). The specimens were then submitted to paired-end whole-metagenomic shotgun sequencing on the Illumina Novaseq 6000 platform (insert size, 300 bp; read length, 150 bp).

Bioinformatic assessment encompassing library construction, prediction of genes, taxonomic annotation and abundance determination was carried out as described in a previous report [2]. In brief, genes were predicted from the assembled contigs with Meta GeneMark prokaryotic hidden Markov model (Version 2.10). A gene library without redundancy was built with Cluster Database at High Identity with Tolerance (CD-HIT, Version 4.5.8) with a sequence identity cutoff of 0.95 and a minimum coverage cutoff of 0.9. Read realignment to the gene library with SOAP2 was carried out utilizing parameters for determining gene abundance levels (− m 200 − x 400 − s 119). Only genes containing 2 or more mapped reads were further assessed. Gene abundance determination was performed by counting reads with normalization to the number of base pairs, and the abundance of genes was calculated by counting the number of reads and normalizing by gene length as previously described [2, 30, 31], and the structure of the formula could be summarized as [${G}_{i}=\frac{{r}_{i}}{{L}_{i}}\bullet \frac{1}{{\sum }_{i}^{n}\frac{{r}_{i}}{{L}_{i}}}$], where the “r” denotes the number of reads and the “L” means the gene length. Using DIAMOND v0.7.9.58, the totality of library genes underwent alignment to the KEGG database (Release 73.1; animal and plant genes excluded). Every protein was assigned to KEGG orthologues utilizing hits with highest scores encompassing ≥ 1 high-score segment pair totaling > 60 hits. By adding up the abundance levels of all genes assigned to the identical property, the abundance of a given KEGG ortholog was determined.

The protein sequences of *yciA*, *tesA*, *tesB*, *menI*, *propionyl CoA transferase*, *CO dehydrogenase acetyl-CoA synthase complex*, and *butyrate acetoacetate CoA transferase* were downloaded from http://www.ncbi.nlm.nih.gov/. The relative abundance levels of enzyme genes and harboring species were obtained by aligning the non-redundant gene library to the sequences with BLASTP v2.6.0 [32]. Firstly, a reference database was conducted based on protein sequences of targeted enzyme genes downloaded from KEGG database. Secondly, enzyme genes were identified by aligning non-redundant genes to the reference database using blastp (parameters, -evalue 1e-5 -outfmt 6 -num_alignments 10). Then, the relative abundance levels of enzyme genes were determined by summing the abundance of non-redundant genes annotated to the same enzyme. At last, taxonomic classification of enzyme genes was executed according to the taxonomic annotation of related genes which were assessed from previous analysis [2] as followed. Genes were aligned to the integrated nr database to assess the taxonomic assignment by using DIAMOND (Version 0.7.9.58, default parameters except that − k 50 − sensitive − e 0.00001) [33]. To distinguish taxonomic groups, the significant matches for each gene, defined by e-values ≤ 10 × e-value of the top hit, were determined and the retained matches were used [34]. The taxonomical level of each gene was determined using the lowest common ancestor − based algorithm implemented with MEGAN (MEtaGenome ANalyzer) [35].

### Statistical analysis

Abundance disparities of KOs, enzymatic genes and harboring species were assessed by the Wilcoxon rank sum test, with Benjamin and Hochberg correction; a q value < 0.05 denoted statistical difference. Venn, Sankey and radar plots were graphed with the R (Version 0.6) packages UpSetR, fmsb and riverplot, respectively.

The least absolute shrinkage and selection operator (LASSO) technique, applied recently in multiple radiomic, genomic and metagenomics reports, was utilized to select the best parameters that could distinguish non-AF control and AF patients. KO, enzyme and species scores were calculated for individual participants by linearly combining the retained parameters with respective coefficients. Internal validation was carried out as previously reported. The average of 500 bootstrapped estimates of optimism was subtracted from the initial (full cohort model) estimate of the AUC and Nagelkerke R2 for obtaining the bootstrap optimism-corrected estimates of performance [5].

The odds ratio (OR) for each KEGG ortholog (k) was determined based on the following formula: OR(k) = [∑s = CTR Ask / ∑s = CTR (∑i ≠ k Asi)] / [∑s = AF Ask / ∑s = AF (∑i ≠ k Asi)], where Ask is the abundance of the KEGG ortholog/module k in specimens [36]. The equation could be generalized as [sum(s) / sum(a)], where “sum(s)” and “sum(a)” are the sums of KO1 and non-KO1 in the AF/CTR group, respectively. The KEGG orthologues were next defined as AF- (Log 2 [OR score] > 0) or CTR- (Log 2 [OR score] < 0) enriched.

## Supplementary Information

**Additional file 1:** Supplementary **Table S1**. The list of SCFA-related KOs.

**Additional file 2:** Supplementary**Table S2**. Spearman correlation among 10 species that harbored synthesis enzyme genes for the SCFAs-related compounds.

**Additional file 3:** Supplementary **Table S3**. Univariate and multivariable logistic regression analyses of discriminative SCFAs-related factors.

**Additional file 4:** Supplementary **Table S4**. The baseline characteristics data of the 100 individuals.

**Additional file 5:** Supplementary **Figure S1**. 125 differential SCFAs-related KOs between non-AF controls and AF patients.

**Additional file 6:** Supplementary**Figure S2**. BCFAs-related synthetic enzyme genes and harboring species in the gut of AF patients

**Additional file 7:** Supplementary** Figure S3**. LASSO analysis based on discriminative SCFAs-related factors.

## Data Availability

All data in the current study are available at European Molecular Biology Laboratory (EMBL) European Nucleotide Archive (ENA) with the BioProject accession code PRJEB28384 [https://www.ebi.ac.uk/ena/data/view/PRJEB28384].

## Abbreviations

SCFAs: Short-chain fatty acids
AF: Atrial fibrillation
KEGG: Kyoto Encyclopedia of Genes and Genomes
KOs: KEGG orthologues
ORs: Odds ratios
AUC: Area under curve
PLS-SEM: Partial least squares structural equation modeling
IDEs: Indirect effects
VAF: Variance accounted for
hsCRP: High-sensitive c reactive protein
NLRP3: NACHT, LRR and PYD domain containing protein 3
CMs: Cardiomyocytes
KI: Knock-in
GPR43: G-protein coupled-receptor-43
LASSO: Least absolute shrinkage and selection operator
EMBL: European Molecular Biology Laboratory
ENA: European Nucleotide Archive
