# Supplementary material for: Altered synthesis of genes associated with short-chain fatty acids in the gut of patients with atrial fibrillation
Source: BMC Genomics. 2021 Aug 31;22:634. doi: 10.1186/s12864-021-07944-0 (PMC8406843; doi:10.1186/s12864-021-07944-0)
Supplement: Supplementary file 3 — Additional file 3: Supplementary Table S3. Univariate and multivariable logistic regression analyses of discriminative SCFAs-related factors. [file 12864_2021_7944_MOESM3_ESM.docx]

**Table S3. Univariate and multivariable logistic regression analysis for predicting AF.**

| **Clinical factor** | **Univariable** | | | **Multivariable** | | |
| --- | --- | --- | --- | --- | --- | --- |
|  | **B** | **P value** | **OR (95% CI)** | **B** | **P value** | **OR (95% CI)** |
| Age | 0.146 | 8E-06 | 1.157 (1.085, 1.234) | 0.115 | 0.07 | 1.121 (0.991, 1.270) |
| BMI | 0.114 | 0.047 | 1.121 (1.001, 1.255) | 0.425 | 0.114 | 1.529 (0.904, 2.587) |
| HTN | 0.000 | 1.000 | 1.000 (0.455, 2.196) | / | | |
| T2DM | 21.477 | 0.999 | 2.126E+9 (0, /) | / | | |
| KO score | -4.426 | 1.1E-05 | 0.012 (0.002, 0.086) | -5.559 | 0.001 | 0.004 (1.54E-04, 0.097) |

**Abbreviations:** OR, odds ratio; CI, confidence interval; BMI, body mass index; HTN, hypertension; T2DM, type 2 diabetes mellitus; KO score =-1.993*(Intercept)+(830.2118*K01752)+(301.6231*K01738)+(6023.7645*K00175) +(955.9364*K03737)+(253.4848*K01006)+(-134.3974*K01653)+(-2850.2412*K01647)+(-2688.7880*K15023), and the formula of KO score was built LASSO analysis.
