# Supplementary material for: Altered synthesis of genes associated with short-chain fatty acids in the gut of patients with atrial fibrillation
Source: BMC Genomics. 2021 Aug 31;22:634. doi: 10.1186/s12864-021-07944-0 (PMC8406843; doi:10.1186/s12864-021-07944-0)
Supplement: Supplementary file 4 — Additional file 4: Supplementary Table S4. The baseline characteristics data of the 100 individuals. [file 12864_2021_7944_MOESM4_ESM.docx]

**Supplementary Table S4. Baseline characteristics of the 100 individuals.**

|  | **AF (n=50)** | **CTR (n=50)** | **P value** |
| --- | --- | --- | --- |
| **Age, years** | 66 (57, 71.25) | 55 (50.5, 57.5) | <0.001 |
| **Male / Female** | 32/18 | 41/9 | 0.043 |
| **Body mass index** | 26.46 (23.79, 28.64) | 24.77 (22.79, 27.62) | 0.112 |
| **Hypertension** | 27 | 27 | / |
| **Diabetes mellitus** | 12 | 0 | 0.0002 |
| **Total cholesterol** | 4.13±1.05 | 4.82±0.96 | 0.001 |
| **Triglyceride** | 1.29 (1.02, 1.88) | 1.06 (0.77, 1.80) | 0.084 |
| **LDL cholesterol** | 2.45 (1.58, 2.93) | 2.3 (1.96, 2.86) | 0.872 |
| **Fasting blood glucose** | 4.95 (4.50, 5.83) | 5.12 (4.56, 5.55) | 0.883 |
| **Creatinine** | 68.5 (60.48, 79.35) | 70 (60, 89.5) | 0.533 |
| **Total bilirubin** | 14 (10.08, 19.5) | 14.7 (11.59, 19.75) | 0.431 |
| **Glutamic-pyruvic transaminase** | 19 (13.75, 28.5) | 19 (12, 25) | 0.185 |

**Abbreviations:** AF, atrial fibrillation; CTR, non-AF control; LDL, low density lipoprotein. Data are presented as mean± SD, or median (interquartile range), as appropriate.
