# Supplementary figures and images for: Altered synthesis of genes associated with short-chain fatty acids in the gut of patients with atrial fibrillation
Source: BMC Genomics. 2021 Aug 31;22:634. doi: 10.1186/s12864-021-07944-0 (PMC8406843; doi:10.1186/s12864-021-07944-0)

125 differed SCFA-related KEGG Orthology (KO) between CTR and AF

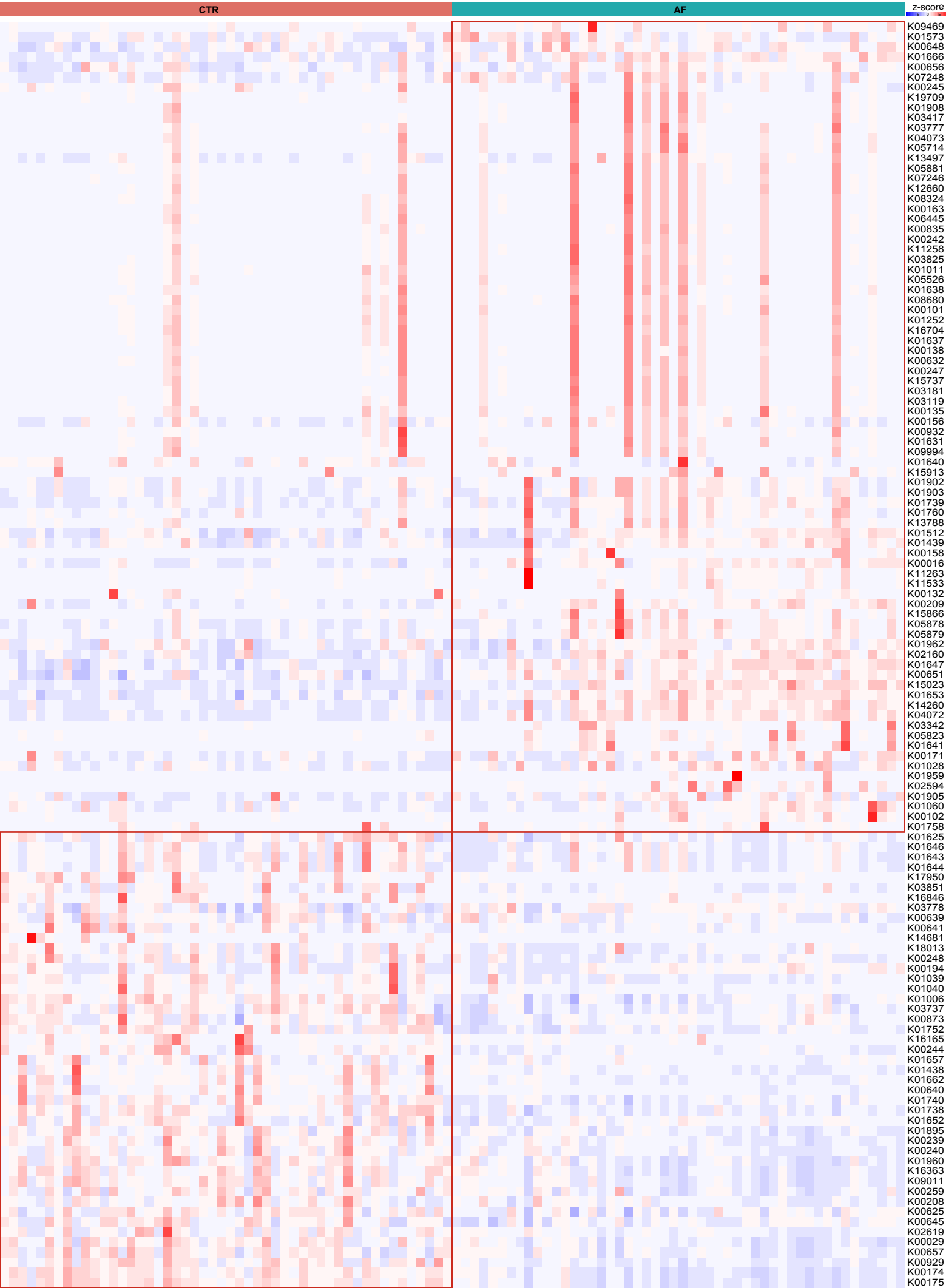

Supplement: Supplementary file 5 — Additional file 5: Supplementary Figure S1. 125 differential SCFAs-related KOs between non-AF controls and AF patients. [file 12864_2021_7944_MOESM5_ESM.pdf]

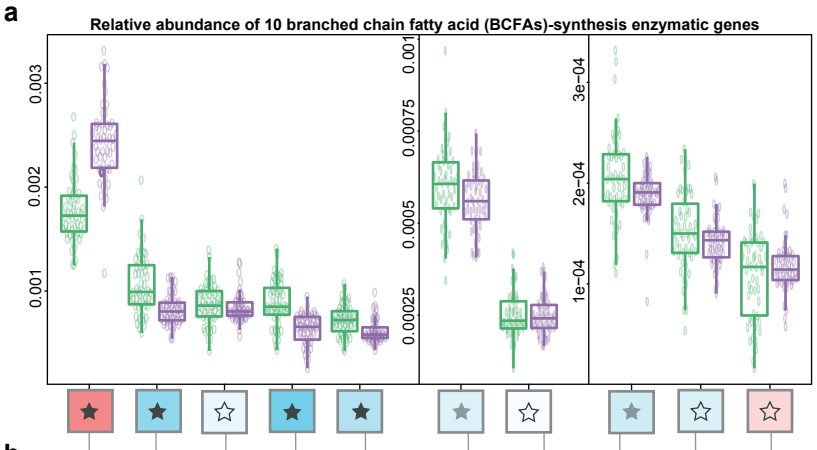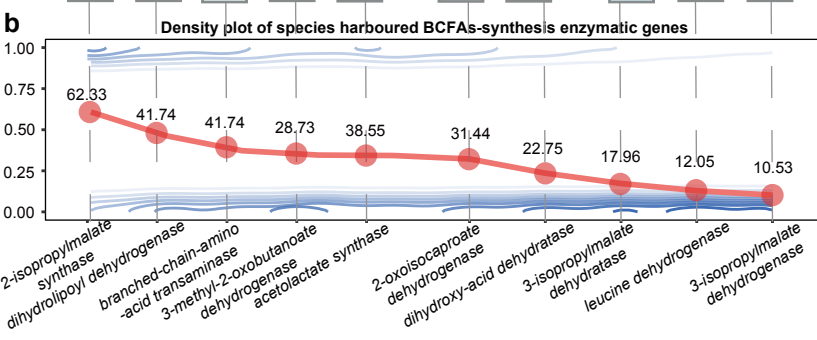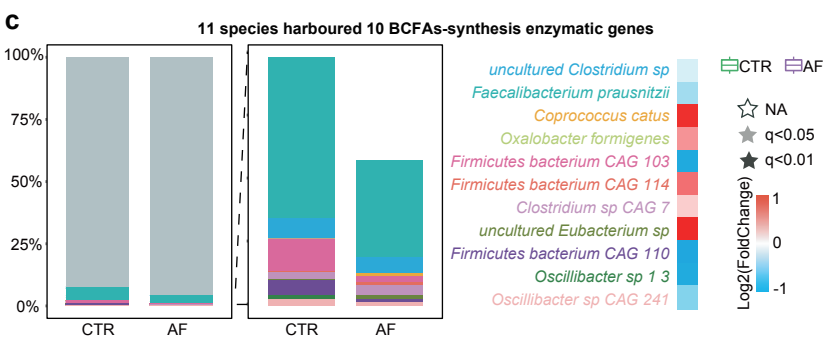

Supplement: Supplementary file 6 — Additional file 6: SupplementaryFigure S2. BCFAs-related synthetic enzyme genes and harboring species in the gut of AF patients [file 12864_2021_7944_MOESM6_ESM.pdf]

**a Species:**

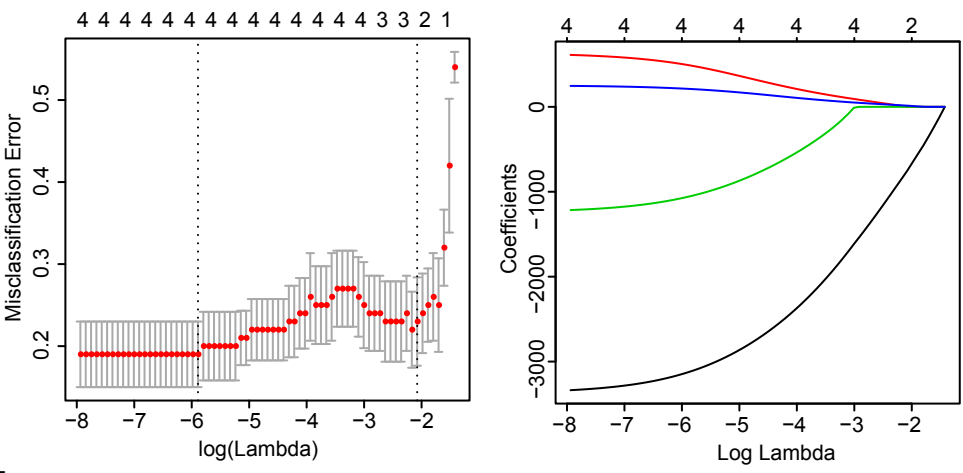

**b Enzyme:**

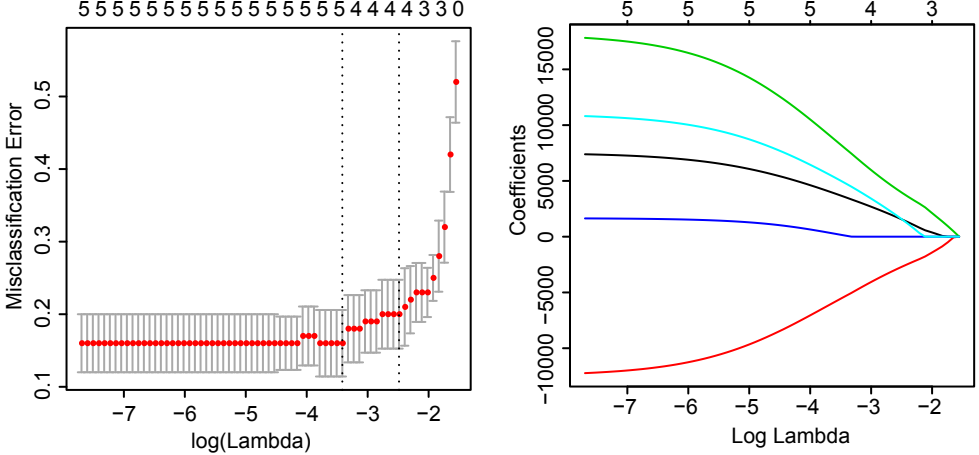

**c KO:**

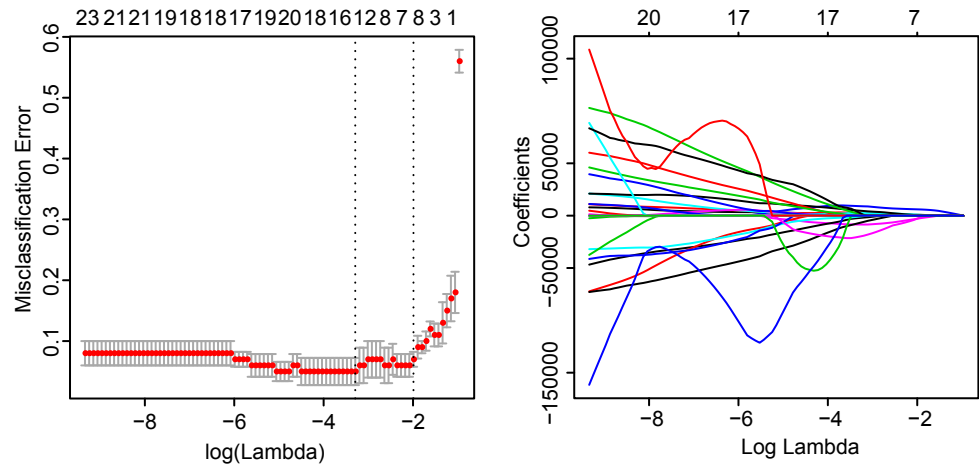

Supplement: Supplementary file 7 — Additional file 7: Supplementary Figure S3. LASSO analysis based on discriminative SCFAs-related factors. [file 12864_2021_7944_MOESM7_ESM.pdf]
